# Supplementary material for: COZOID: contact zone identifier for visual analysis of protein-protein interactions
Source: BMC Bioinformatics. 2018 Apr 6;19:125. doi: 10.1186/s12859-018-2113-6 (PMC5889581; doi:10.1186/s12859-018-2113-6)
Supplement: Supplementary file 2 — Software build. Executable binary file of the software tool. (ZIP 118,784 kb) [file 12859_2018_2113_MOESM2_ESM.zip › cozoid/java/docs/beansbinding-1.2.1-doc.zip!/org/jdesktop/el/class-use/FunctionMapper.html]

Uses of Class org.jdesktop.el.FunctionMapper (Beans Binding)


---


|  |  |  |
| --- | --- | --- |
| **Overview** \| **Package** \| **Class** \| **Use** \| **Tree** \| **Deprecated** \| **Index** \| **Help** | |  |
| PREV   NEXT | **FRAMES**    **NO FRAMES**     **All Classes** |  |


---


## **Uses of Class org.jdesktop.el.FunctionMapper**

| Packages that use FunctionMapper | |
| --- | --- |
| **org.jdesktop.el** | Customized version of EL for Beans Binding; not for general use. |
| **org.jdesktop.el.impl** | Customized version of EL for Beans Binding; not for general use. |
| **org.jdesktop.el.impl.lang** | Customized version of EL for Beans Binding; not for general use. |

Uses of FunctionMapper in org.jdesktop.el

| Methods in org.jdesktop.el that return FunctionMapper | |
| --- | --- |
| `abstract  FunctionMapper` | `ELContext.getFunctionMapper()`             Retrieves the `FunctionMapper` associated with this `ELContext`. |

Uses of FunctionMapper in org.jdesktop.el.impl

Constructors in org.jdesktop.el.impl with parameters of type FunctionMapper

`MethodExpressionImpl(java.lang.String expr,
Node node,
FunctionMapper fnMapper,
VariableMapper varMapper,
java.lang.Class expectedType,
java.lang.Class[] paramTypes)`

`ValueExpressionImpl(java.lang.String expr,
Node node,
FunctionMapper fnMapper,
VariableMapper varMapper,
java.lang.Class expectedType)`

Uses of FunctionMapper in org.jdesktop.el.impl.lang

| Subclasses of FunctionMapper in org.jdesktop.el.impl.lang | |
| --- | --- |
| `class` | `FunctionMapperFactory` |
| `class` | `FunctionMapperImpl` |

| Fields in org.jdesktop.el.impl.lang declared as FunctionMapper | |
| --- | --- |
| `protected  FunctionMapper` | `FunctionMapperFactory.target` |

| Methods in org.jdesktop.el.impl.lang that return FunctionMapper | |
| --- | --- |
| `FunctionMapper` | `FunctionMapperFactory.create()` |
| `FunctionMapper` | `EvaluationContext.getFunctionMapper()` |

Constructors in org.jdesktop.el.impl.lang with parameters of type FunctionMapper

`EvaluationContext(ELContext elContext,
FunctionMapper fnMapper,
VariableMapper varMapper,
Expression expression)`

`EvaluationContext(ELContext elContext,
FunctionMapper fnMapper,
VariableMapper varMapper,
Expression expression,
boolean trackResolvedProperties)`

`FunctionMapperFactory(FunctionMapper mapper)`

---


|  |  |  |
| --- | --- | --- |
| **Overview** \| **Package** \| **Class** \| **Use** \| **Tree** \| **Deprecated** \| **Index** \| **Help** | |  |
| PREV   NEXT | **FRAMES**    **NO FRAMES**     **All Classes** |  |


---
